# Methyl 11-hy­droxy-9-[1-(4-meth­oxy­phen­yl)-4-oxo-3-phen­oxy­azetidin-2-yl]-18-oxo-10-oxa-2-aza­penta­cyclo­[9.7.0.0^1,8^.0^2,6^.0^12,17^]octa­deca-12(17),13,15-triene-8-carboxyl­ate

**DOI:** 10.1107/S1600536812027341

**Published:** 2012-06-23

**Authors:** S. Sundaramoorthy, R. Rajesh, R. Raghunathan, D. Velmurugan

**Affiliations:** a Centre of Advanced Study in Crystallography and Biophysics, University of Madras, Guindy Campus, Chennai 600 025, India; b Department of Organic Chemistry, University of Madras, Guindy Campus, Chennai 600 025, India

## Abstract

In the title compound, C_34_H_32_N_2_O_8_, one of the pyrrolidine rings in the pyrrolizidine ring system adopts a twist conformation, whereas the other ring adopts an envelope conformation (C atom as flap). The five-membered ring in the indene ring system and the fused furan ring also adopt envelope conformations (C and O atoms as flaps, respectively). The β-lactam ring makes dihedral angles of 23.41 (2) and 25.98 (2)°, respectively, with the attached meth­oxy­phenyl and phen­oxy rings. The mol­ecular conformation is stabilized by an intra­molecular O—H⋯N hydrogen bond, generating an *S*(5) motif. In the crystal, mol­ecules are linked into *C*(12) chains running along the *a* axis by C—H⋯O hydrogen bonds. The structure is further consolidated by weak inter­molecular C—H⋯π and π–π inter­actions [centroid–centroid distance = 3.7987 (14) Å].

## Related literature

For general background to β-lactams, see: Banik & Becker (2000 ▶); Brakhage (1998 ▶). For a related structure, see: Arun *et al.* (2003 ▶). For hydrogen-bond motifs, see: Bernstein *et al.* (1995 ▶).
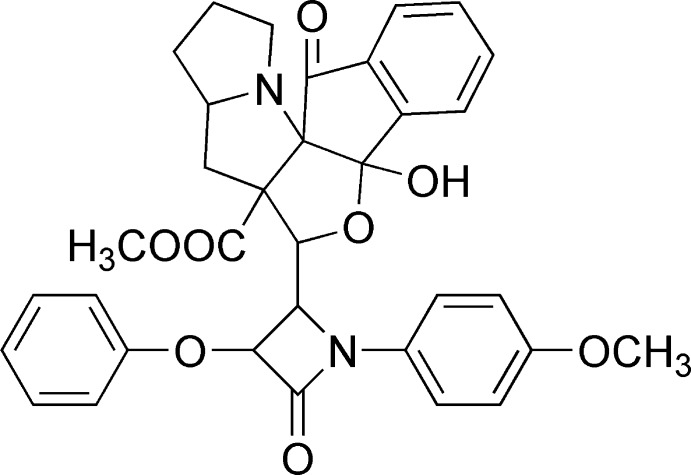


## Experimental

### 

#### Crystal data

C_34_H_32_N_2_O_8_
*M* *_r_* = 596.62
Monoclinic, 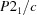

*a* = 11.4251 (13) Å
*b* = 7.8362 (8) Å
*c* = 32.041 (4) Å
β = 91.313 (8)°
*V* = 2867.9 (6) Å^3^
*Z* = 4
Mo *K*α radiation
μ = 0.10 mm^−1^
*T* = 293 K
0.25 × 0.22 × 0.2 mm

#### Data collection

Bruker SMART APEXII area-detector diffractometer
Absorption correction: multi-scan (*SADABS*; Bruker, 2008 ▶) *T*_min_ = 0.976, *T*_max_ = 0.980
27667 measured reflections
7104 independent reflections
3251 reflections with *I* > 2σ(*I*)
*R*_int_ = 0.083

#### Refinement

*R*[*F* ^2^ > 2σ(*F* ^2^)] = 0.054
*wR*(*F* ^2^) = 0.141
*S* = 0.99
7104 reflections
400 parameters
H-atom parameters constrained
Δρ_max_ = 0.26 e Å^−3^
Δρ_min_ = −0.20 e Å^−3^

### 

Data collection: *APEX2* (Bruker, 2008 ▶); cell refinement: *SAINT* (Bruker, 2008 ▶); data reduction: *SAINT*; program(s) used to solve structure: *SHELXS97* (Sheldrick, 2008 ▶); program(s) used to refine structure: *SHELXL97* (Sheldrick, 2008 ▶); molecular graphics: *ORTEP-3* (Farrugia, 1997 ▶); software used to prepare material for publication: *SHELXL97* and *PLATON* (Spek, 2009 ▶).

## Supplementary Material

Crystal structure: contains datablock(s) global, I. DOI: 10.1107/S1600536812027341/pv2560sup1.cif

Structure factors: contains datablock(s) I. DOI: 10.1107/S1600536812027341/pv2560Isup2.hkl

Supplementary material file. DOI: 10.1107/S1600536812027341/pv2560Isup3.cml

Additional supplementary materials:  crystallographic information; 3D view; checkCIF report

## Figures and Tables

**Table 1:** Hydrogen-bond geometry (Å, °) *Cg*7 is the centroid of the C11–C16 ring.

*D*—H⋯*A*	*D*—H	H⋯*A*	*D*⋯*A*	*D*—H⋯*A*
O5—H5*A*⋯N2	0.82	2.10	2.638 (2)	122
C15—H15⋯O6^i^	0.93	2.56	3.231 (3)	129
C12—H12⋯*Cg*7^ii^	0.93	2.95	3.775 (3)	149

Symmetry codes: (i) 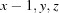; (ii) 
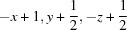
.

**Fig. 1.**
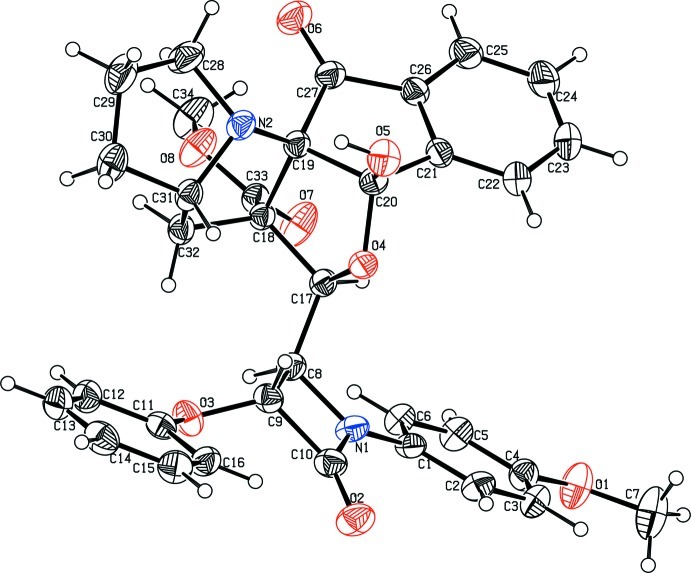
A perspective view of the molecule showing the thermal ellipsoids drawn at the 30% probability level.

**Fig. 2.**
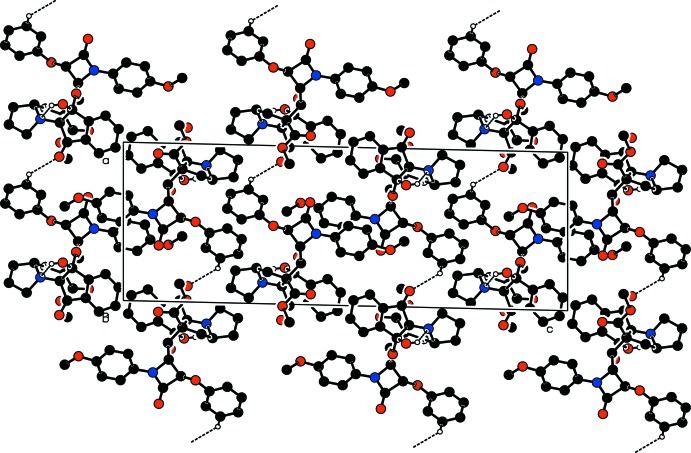
C—H···O and O—H···N interactions (dotted lines) in the crystal structure of the title compound viewed down the b axis.

## supplementary crystallographic information

### Comment

The role of β-lactam antibiotics is well known (Banik & Becker, 2000). The most commonly used β-lactam antibiotics for the therapy of infectious diseases are penicillin and cephalosporin (Brakhage, 1998). In view of potential applications, the crystal structure determination of the title β-lactam derivative was carried out which is reported in this article.

In the title compound (Fig. 1), the β-lactam ring makes dihedral angle of 31.36 (1)° with the indene ring system. The maximum deviation of indene ring system is 0.212 (2) Å for atom C19. The β-lactam ring makes dihedral angles 23.41 (15) and 25.98 (15)°, respectively, with the attached methoxyphenyl and phenoxy rings. The carboxylate group adopts an extended conformation with thetorsion angle C18—C33—O8—C34 = 171.9 (2)°. The five-membered ring (C19/C20/C21/C26/C27) in the inden ring system and the fused furan ring (O4/C17—C20) adopt envelope conformations, with C19 and O4 atoms deviating by 0.135 (2) and -0.221 (2) Å, respectively, from the planes formed by the remaining atoms of the rings. In the pyrrolizidine ring system, the pyrrolidine ring (C18/C19/N2/C31/C32) adopts a C31-envelope conformation, with C31 deviating by 0.216 (2) Å from the remaining ring atoms. The bond lengths and bond angles in the title compound agree with the corresponding bond lengths and angles reported for a closely related compound (Arun *et al.*, (2003).

The molecular structure of the title compound is stabilized by a strong O5—H5A···N2 hydrogen bond, generating an S(5) motif (Bernstein *et al.*, 1995). The H-atom bonded to C15 is involved in hydrogen bonding with atom O6 forming a C(12) chain running along the *a* axis (Tab. 1 & Fig. 2). A weak intermolecular C—H···π interaction involving the C12—H12 group and the C1—C16 phenoxy ring is also observed.

### Experimental

To a reaction mixture of 2-(hydroxy(1-(4-methoxyphenyl)-4-oxo-3-phenoxyazetidin -2-yl)methyl)acrylate (1 mmol), ninhydrine (1.1 mmol) and proline (1.1 mmol) were refluxed in methanol until completion of the reaction was evidenced by TLC analysis. After completion of the reaction the solvent was evaporated under reduced pressure. The crude reaction mixture was dissolved in dichloromethane and washed with water followed by brine solution. The organic layer was separated and dried over sodium sulfate. Filtering and evaporation of the organic solvent under reduced pressure were carried out. The product was separated by column chromatography using hexane and ethyl acetate (3: 7) as an eluent to give colorless solid. The product was dissolved in chloroform and heated for two minutes. The resulting solution was subjected to crystallization by slow evaporation of the solvent resulting in single crystals of the title compound suitable for XRD studies.

### Refinement

The H atoms were positioned geometrically with O—H = 0.82 Å and C—H = 0.93, 0.96, 0.97 and 0.98 Å for aryl, methyl, methylene and methyne H atoms, respectively, and constrained to ride on their parent atoms, with *U*_iso_(H) = 1.5*U*_eq_(methyl C/O) or 1.2*U*_eq_(non-methyl C).

### Crystal data

**Table:**

C_34_H_32_N_2_O_8_	*F*(000) = 1256
*M**_r_* = 596.62	*D*_x_ = 1.382 Mg m^−^^3^
Monoclinic, *P*2_1_/*c*	Mo *K*α radiation, λ = 0.71073 Å
Hall symbol: -P 2ybc	Cell parameters from 1035 reflections
*a* = 11.4251 (13) Å	θ = 1.3–28.3°
*b* = 7.8362 (8) Å	µ = 0.10 mm^−^^1^
*c* = 32.041 (4) Å	*T* = 293 K
β = 91.313 (8)°	Block, colourless
*V* = 2867.9 (6) Å^3^	0.25 × 0.22 × 0.2 mm
*Z* = 4	

### Data collection

**Table:**

Bruker SMART APEXII area-detector diffractometer	7104 independent reflections
Radiation source: fine-focus sealed tube	3251 reflections with *I* > 2σ(*I*)
Graphite monochromator	*R*_int_ = 0.083
ω and φ scans	θ_max_ = 28.3°, θ_min_ = 1.3°
Absorption correction: multi-scan (*SADABS*; Bruker, 2008)	*h* = −15→15
*T*_min_ = 0.976, *T*_max_ = 0.980	*k* = −10→10
27667 measured reflections	*l* = −42→42

### Refinement

**Table:**

Refinement on *F*^2^	Primary atom site location: structure-invariant direct methods
Least-squares matrix: full	Secondary atom site location: difference Fourier map
*R*[*F*^2^ > 2σ(*F*^2^)] = 0.054	Hydrogen site location: inferred from neighbouring sites
*wR*(*F*^2^) = 0.141	H-atom parameters constrained
*S* = 0.99	*w* = 1/[σ^2^(*F*_o_^2^) + (0.0537*P*)^2^] where *P* = (*F*_o_^2^ + 2*F*_c_^2^)/3
7104 reflections	(Δ/σ)_max_ < 0.001
400 parameters	Δρ_max_ = 0.26 e Å^−^^3^
0 restraints	Δρ_min_ = −0.20 e Å^−^^3^

### Special details

**Table:**

Geometry. All e.s.d.'s (except the e.s.d. in the dihedral angle between two l.s. planes) are estimated using the full covariance matrix. The cell e.s.d.'s are taken into account individually in the estimation of e.s.d.'s in distances, angles and torsion angles; correlations between e.s.d.'s in cell parameters are only used when they are defined by crystal symmetry. An approximate (isotropic) treatment of cell e.s.d.'s is used for estimating e.s.d.'s involving l.s. planes.
Refinement. Refinement of *F*^2^ against ALL reflections. The weighted *R*-factor *wR* and goodness of fit *S* are based on *F*^2^, conventional *R*-factors *R* are based on *F*, with *F* set to zero for negative *F*^2^. The threshold expression of *F*^2^ > σ(*F*^2^) is used only for calculating *R*-factors(gt) *etc*. and is not relevant to the choice of reflections for refinement. *R*-factors based on *F*^2^ are statistically about twice as large as those based on *F*, and *R*- factors based on ALL data will be even larger.

### Fractional atomic coordinates and isotropic or equivalent isotropic displacement parameters (Å^2^)

**Table:**

	*x*	*y*	*z*	*U*_iso_*/*U*_eq_	
C1	0.5692 (2)	0.7918 (3)	0.02316 (7)	0.0411 (6)	
C2	0.4969 (2)	0.7168 (3)	−0.00647 (7)	0.0494 (6)	
H2	0.4337	0.6513	0.0018	0.059*	
C3	0.5175 (2)	0.7380 (3)	−0.04839 (8)	0.0523 (7)	
H3	0.4682	0.6878	−0.0683	0.063*	
C4	0.6120 (2)	0.8343 (3)	−0.06043 (7)	0.0484 (6)	
C5	0.6846 (2)	0.9091 (3)	−0.03100 (7)	0.0513 (7)	
H5	0.7482	0.9738	−0.0394	0.062*	
C6	0.6638 (2)	0.8886 (3)	0.01066 (7)	0.0469 (6)	
H6	0.7130	0.9396	0.0305	0.056*	
C7	0.5862 (3)	0.7665 (4)	−0.13225 (9)	0.0926 (11)	
H7A	0.5981	0.6474	−0.1266	0.139*	
H7B	0.6184	0.7943	−0.1588	0.139*	
H7C	0.5038	0.7911	−0.1328	0.139*	
C8	0.6332 (2)	0.7868 (3)	0.10200 (7)	0.0416 (6)	
H8	0.6557	0.9058	0.1071	0.050*	
C9	0.53063 (19)	0.7311 (3)	0.12942 (7)	0.0420 (6)	
H9	0.5417	0.6144	0.1397	0.050*	
C10	0.4546 (2)	0.7338 (3)	0.08915 (8)	0.0478 (6)	
C11	0.4395 (2)	0.7776 (3)	0.19371 (7)	0.0412 (6)	
C12	0.4698 (2)	0.8218 (3)	0.23396 (8)	0.0511 (6)	
H12	0.5336	0.8928	0.2394	0.061*	
C13	0.4048 (2)	0.7598 (3)	0.26613 (8)	0.0564 (7)	
H13	0.4247	0.7892	0.2935	0.068*	
C14	0.3106 (2)	0.6547 (3)	0.25828 (8)	0.0563 (7)	
H14	0.2671	0.6127	0.2802	0.068*	
C15	0.2813 (2)	0.6123 (3)	0.21807 (8)	0.0542 (7)	
H15	0.2174	0.5412	0.2127	0.065*	
C16	0.3446 (2)	0.6730 (3)	0.18552 (7)	0.0478 (6)	
H16	0.3239	0.6441	0.1582	0.057*	
C17	0.73901 (19)	0.6701 (3)	0.10020 (7)	0.0381 (5)	
H17	0.7718	0.6788	0.0723	0.046*	
C18	0.83833 (19)	0.7027 (3)	0.13279 (6)	0.0355 (5)	
C19	0.88557 (19)	0.5159 (3)	0.14320 (6)	0.0376 (5)	
C20	0.8029 (2)	0.3992 (3)	0.11556 (7)	0.0403 (6)	
C21	0.8728 (2)	0.3585 (3)	0.07798 (7)	0.0412 (6)	
C22	0.8357 (2)	0.2882 (3)	0.04033 (8)	0.0530 (7)	
H22	0.7571	0.2629	0.0352	0.064*	
C23	0.9177 (3)	0.2571 (3)	0.01084 (8)	0.0632 (8)	
H23	0.8940	0.2103	−0.0147	0.076*	
C24	1.0348 (3)	0.2935 (4)	0.01796 (8)	0.0647 (8)	
H24	1.0885	0.2691	−0.0026	0.078*	
C25	1.0732 (2)	0.3655 (3)	0.05511 (8)	0.0552 (7)	
H25	1.1518	0.3913	0.0600	0.066*	
C26	0.9898 (2)	0.3977 (3)	0.08485 (7)	0.0428 (6)	
C27	1.0082 (2)	0.4737 (3)	0.12642 (7)	0.0446 (6)	
C28	0.9643 (2)	0.5013 (4)	0.21783 (7)	0.0655 (8)	
H28A	1.0137	0.4006	0.2184	0.079*	
H28B	1.0118	0.6001	0.2114	0.079*	
C29	0.9052 (3)	0.5235 (4)	0.25785 (8)	0.0727 (9)	
H29A	0.9546	0.5866	0.2774	0.087*	
H29B	0.8869	0.4135	0.2700	0.087*	
C30	0.7948 (2)	0.6211 (4)	0.24787 (7)	0.0576 (7)	
H30A	0.8036	0.7400	0.2558	0.069*	
H30B	0.7293	0.5730	0.2626	0.069*	
C31	0.7756 (2)	0.6046 (3)	0.20060 (6)	0.0432 (6)	
H31	0.6970	0.5608	0.1941	0.052*	
C32	0.7991 (2)	0.7655 (3)	0.17556 (6)	0.0424 (6)	
H32A	0.8600	0.8335	0.1891	0.051*	
H32B	0.7287	0.8341	0.1727	0.051*	
C33	0.9311 (2)	0.8179 (3)	0.11405 (7)	0.0413 (6)	
C34	1.1133 (2)	0.9561 (4)	0.12607 (8)	0.0693 (8)	
H34A	1.1559	0.8824	0.1079	0.104*	
H34B	1.1632	0.9908	0.1490	0.104*	
H34C	1.0873	1.0551	0.1108	0.104*	
N1	0.55049 (16)	0.7704 (3)	0.06619 (6)	0.0451 (5)	
N2	0.86574 (17)	0.4818 (2)	0.18691 (5)	0.0433 (5)	
O1	0.64164 (18)	0.8632 (2)	−0.10114 (5)	0.0725 (6)	
O2	0.35322 (16)	0.7067 (2)	0.07968 (5)	0.0623 (5)	
O3	0.50681 (14)	0.8443 (2)	0.16232 (5)	0.0511 (4)	
O4	0.70046 (13)	0.49763 (19)	0.10630 (4)	0.0429 (4)	
O5	0.76948 (16)	0.2530 (2)	0.13645 (5)	0.0549 (5)	
H5A	0.7789	0.2671	0.1617	0.082*	
O6	1.10093 (16)	0.4957 (3)	0.14436 (5)	0.0694 (6)	
O7	0.93303 (17)	0.8573 (3)	0.07831 (5)	0.0721 (6)	
O8	1.01251 (15)	0.8656 (2)	0.14184 (5)	0.0608 (5)	

### Atomic displacement parameters (Å^2^)

**Table:**

	*U*^11^	*U*^22^	*U*^33^	*U*^12^	*U*^13^	*U*^23^
C1	0.0358 (14)	0.0458 (14)	0.0416 (14)	0.0071 (11)	−0.0018 (11)	0.0055 (11)
C2	0.0393 (15)	0.0575 (16)	0.0512 (15)	−0.0035 (12)	−0.0038 (12)	0.0050 (12)
C3	0.0522 (18)	0.0579 (17)	0.0462 (15)	−0.0057 (13)	−0.0102 (13)	−0.0054 (12)
C4	0.0559 (18)	0.0500 (15)	0.0392 (14)	0.0028 (13)	0.0005 (12)	0.0003 (11)
C5	0.0516 (17)	0.0559 (16)	0.0464 (15)	−0.0085 (13)	0.0000 (13)	0.0057 (12)
C6	0.0455 (16)	0.0530 (16)	0.0419 (14)	−0.0050 (12)	−0.0043 (12)	0.0017 (12)
C7	0.129 (3)	0.099 (3)	0.0503 (18)	−0.027 (2)	−0.0015 (19)	−0.0174 (17)
C8	0.0378 (14)	0.0492 (15)	0.0378 (12)	0.0021 (11)	0.0012 (11)	0.0019 (11)
C9	0.0383 (15)	0.0469 (14)	0.0410 (13)	0.0012 (11)	0.0075 (11)	0.0005 (11)
C10	0.0393 (17)	0.0534 (16)	0.0508 (15)	0.0046 (12)	0.0038 (13)	0.0019 (12)
C11	0.0358 (15)	0.0427 (14)	0.0455 (14)	0.0051 (11)	0.0087 (11)	0.0009 (11)
C12	0.0457 (16)	0.0552 (16)	0.0524 (16)	−0.0035 (12)	−0.0003 (13)	−0.0113 (13)
C13	0.065 (2)	0.0616 (18)	0.0421 (15)	0.0095 (15)	−0.0004 (14)	−0.0056 (13)
C14	0.0593 (19)	0.0568 (17)	0.0535 (17)	0.0075 (14)	0.0157 (14)	0.0056 (13)
C15	0.0406 (16)	0.0626 (18)	0.0599 (17)	−0.0058 (13)	0.0073 (13)	−0.0004 (14)
C16	0.0370 (15)	0.0630 (16)	0.0434 (14)	0.0010 (12)	−0.0007 (12)	−0.0036 (12)
C17	0.0344 (14)	0.0468 (14)	0.0331 (12)	0.0000 (11)	0.0019 (10)	−0.0011 (10)
C18	0.0333 (13)	0.0406 (13)	0.0327 (11)	−0.0010 (10)	0.0012 (10)	−0.0024 (10)
C19	0.0360 (14)	0.0441 (14)	0.0327 (12)	0.0005 (10)	0.0012 (10)	−0.0017 (10)
C20	0.0357 (14)	0.0424 (14)	0.0428 (13)	−0.0022 (11)	−0.0003 (11)	−0.0013 (11)
C21	0.0464 (16)	0.0374 (13)	0.0399 (13)	0.0038 (11)	0.0027 (11)	−0.0030 (10)
C22	0.0586 (18)	0.0508 (16)	0.0494 (15)	0.0017 (13)	−0.0016 (14)	−0.0113 (12)
C23	0.072 (2)	0.070 (2)	0.0471 (16)	0.0051 (16)	−0.0005 (15)	−0.0192 (13)
C24	0.069 (2)	0.078 (2)	0.0472 (16)	0.0126 (16)	0.0133 (15)	−0.0088 (14)
C25	0.0470 (17)	0.0676 (18)	0.0515 (16)	0.0088 (13)	0.0082 (13)	−0.0012 (13)
C26	0.0430 (16)	0.0470 (15)	0.0385 (13)	0.0078 (11)	0.0036 (11)	−0.0010 (11)
C27	0.0363 (15)	0.0523 (15)	0.0451 (14)	0.0018 (11)	−0.0019 (12)	−0.0011 (11)
C28	0.0622 (19)	0.092 (2)	0.0416 (15)	0.0089 (16)	−0.0119 (13)	0.0089 (15)
C29	0.096 (3)	0.078 (2)	0.0435 (16)	−0.0044 (18)	−0.0176 (15)	−0.0007 (14)
C30	0.063 (2)	0.0757 (19)	0.0339 (13)	−0.0116 (15)	0.0076 (12)	−0.0020 (13)
C31	0.0398 (15)	0.0565 (15)	0.0334 (12)	−0.0062 (12)	0.0045 (11)	−0.0040 (11)
C32	0.0431 (15)	0.0503 (15)	0.0340 (12)	−0.0014 (11)	0.0034 (10)	−0.0055 (10)
C33	0.0427 (15)	0.0446 (14)	0.0364 (13)	−0.0013 (11)	0.0007 (11)	−0.0020 (11)
C34	0.0558 (19)	0.088 (2)	0.0643 (18)	−0.0359 (16)	0.0021 (14)	0.0101 (16)
N1	0.0325 (12)	0.0625 (14)	0.0403 (11)	0.0034 (9)	−0.0006 (9)	0.0083 (9)
N2	0.0451 (13)	0.0527 (12)	0.0320 (10)	0.0002 (10)	−0.0013 (9)	0.0025 (9)
O1	0.0945 (16)	0.0825 (14)	0.0404 (10)	−0.0238 (11)	0.0005 (10)	−0.0019 (9)
O2	0.0365 (12)	0.0868 (14)	0.0636 (12)	−0.0041 (10)	0.0012 (9)	0.0034 (10)
O3	0.0526 (11)	0.0489 (10)	0.0525 (10)	−0.0027 (8)	0.0174 (9)	−0.0059 (8)
O4	0.0334 (9)	0.0449 (10)	0.0502 (9)	−0.0012 (7)	−0.0027 (7)	−0.0069 (8)
O5	0.0636 (12)	0.0470 (11)	0.0543 (10)	−0.0104 (9)	0.0041 (10)	0.0032 (8)
O6	0.0409 (12)	0.1050 (16)	0.0618 (12)	0.0020 (11)	−0.0062 (9)	−0.0172 (11)
O7	0.0787 (15)	0.0973 (15)	0.0402 (11)	−0.0351 (11)	−0.0028 (9)	0.0131 (10)
O8	0.0526 (12)	0.0825 (13)	0.0471 (10)	−0.0282 (10)	−0.0061 (9)	0.0101 (9)

### Geometric parameters (Å, º)

**Table:**

C1—C2	1.376 (3)	C18—C19	1.593 (3)
C1—C6	1.387 (3)	C19—N2	1.449 (3)
C1—N1	1.410 (3)	C19—C27	1.547 (3)
C2—C3	1.379 (3)	C19—C20	1.572 (3)
C2—H2	0.9300	C20—O5	1.385 (3)
C3—C4	1.379 (3)	C20—O4	1.427 (3)
C3—H3	0.9300	C20—C21	1.495 (3)
C4—C5	1.373 (3)	C21—C26	1.384 (3)
C4—O1	1.374 (3)	C21—C22	1.384 (3)
C5—C6	1.371 (3)	C22—C23	1.368 (3)
C5—H5	0.9300	C22—H22	0.9300
C6—H6	0.9300	C23—C24	1.381 (4)
C7—O1	1.393 (3)	C23—H23	0.9300
C7—H7A	0.9600	C24—C25	1.380 (3)
C7—H7B	0.9600	C24—H24	0.9300
C7—H7C	0.9600	C25—C26	1.386 (3)
C8—N1	1.475 (3)	C25—H25	0.9300
C8—C17	1.518 (3)	C26—C27	1.469 (3)
C8—C9	1.543 (3)	C27—O6	1.206 (3)
C8—H8	0.9800	C28—C29	1.474 (4)
C9—O3	1.409 (3)	C28—N2	1.491 (3)
C9—C10	1.539 (3)	C28—H28A	0.9700
C9—H9	0.9800	C28—H28B	0.9700
C10—O2	1.209 (3)	C29—C30	1.503 (4)
C10—N1	1.364 (3)	C29—H29A	0.9700
C11—C12	1.372 (3)	C29—H29B	0.9700
C11—C16	1.379 (3)	C30—C31	1.531 (3)
C11—O3	1.383 (3)	C30—H30A	0.9700
C12—C13	1.373 (3)	C30—H30B	0.9700
C12—H12	0.9300	C31—N2	1.483 (3)
C13—C14	1.374 (3)	C31—C32	1.521 (3)
C13—H13	0.9300	C31—H31	0.9800
C14—C15	1.365 (3)	C32—H32A	0.9700
C14—H14	0.9300	C32—H32B	0.9700
C15—C16	1.368 (3)	C33—O7	1.186 (2)
C15—H15	0.9300	C33—O8	1.327 (3)
C16—H16	0.9300	C34—O8	1.453 (3)
C17—O4	1.436 (3)	C34—H34A	0.9600
C17—C18	1.546 (3)	C34—H34B	0.9600
C17—H17	0.9800	C34—H34C	0.9600
C18—C33	1.526 (3)	O5—H5A	0.8200
C18—C32	1.533 (3)		
			
C2—C1—C6	119.6 (2)	O5—C20—C21	111.76 (19)
C2—C1—N1	121.4 (2)	O4—C20—C21	113.51 (18)
C6—C1—N1	119.0 (2)	O5—C20—C19	112.18 (17)
C1—C2—C3	120.5 (2)	O4—C20—C19	106.39 (17)
C1—C2—H2	119.8	C21—C20—C19	104.65 (18)
C3—C2—H2	119.8	C26—C21—C22	120.2 (2)
C2—C3—C4	119.4 (2)	C26—C21—C20	110.86 (19)
C2—C3—H3	120.3	C22—C21—C20	128.9 (2)
C4—C3—H3	120.3	C23—C22—C21	118.2 (3)
C5—C4—O1	115.1 (2)	C23—C22—H22	120.9
C5—C4—C3	120.4 (2)	C21—C22—H22	120.9
O1—C4—C3	124.6 (2)	C22—C23—C24	121.7 (2)
C6—C5—C4	120.2 (2)	C22—C23—H23	119.2
C6—C5—H5	119.9	C24—C23—H23	119.2
C4—C5—H5	119.9	C25—C24—C23	121.0 (3)
C5—C6—C1	120.0 (2)	C25—C24—H24	119.5
C5—C6—H6	120.0	C23—C24—H24	119.5
C1—C6—H6	120.0	C24—C25—C26	117.2 (3)
O1—C7—H7A	109.5	C24—C25—H25	121.4
O1—C7—H7B	109.5	C26—C25—H25	121.4
H7A—C7—H7B	109.5	C21—C26—C25	121.8 (2)
O1—C7—H7C	109.5	C21—C26—C27	110.6 (2)
H7A—C7—H7C	109.5	C25—C26—C27	127.6 (2)
H7B—C7—H7C	109.5	O6—C27—C26	126.5 (2)
N1—C8—C17	114.50 (18)	O6—C27—C19	126.6 (2)
N1—C8—C9	86.40 (17)	C26—C27—C19	106.85 (19)
C17—C8—C9	117.85 (19)	C29—C28—N2	103.6 (2)
N1—C8—H8	111.9	C29—C28—H28A	111.0
C17—C8—H8	111.9	N2—C28—H28A	111.0
C9—C8—H8	111.9	C29—C28—H28B	111.0
O3—C9—C10	120.2 (2)	N2—C28—H28B	111.0
O3—C9—C8	114.22 (19)	H28A—C28—H28B	109.0
C10—C9—C8	86.58 (17)	C28—C29—C30	105.9 (2)
O3—C9—H9	111.2	C28—C29—H29A	110.6
C10—C9—H9	111.2	C30—C29—H29A	110.6
C8—C9—H9	111.2	C28—C29—H29B	110.6
O2—C10—N1	132.7 (2)	C30—C29—H29B	110.6
O2—C10—C9	136.7 (2)	H29A—C29—H29B	108.7
N1—C10—C9	90.6 (2)	C29—C30—C31	105.5 (2)
C12—C11—C16	120.6 (2)	C29—C30—H30A	110.6
C12—C11—O3	117.1 (2)	C31—C30—H30A	110.6
C16—C11—O3	122.3 (2)	C29—C30—H30B	110.6
C11—C12—C13	119.1 (2)	C31—C30—H30B	110.6
C11—C12—H12	120.4	H30A—C30—H30B	108.8
C13—C12—H12	120.4	N2—C31—C32	104.47 (18)
C12—C13—C14	120.6 (2)	N2—C31—C30	105.24 (19)
C12—C13—H13	119.7	C32—C31—C30	115.39 (19)
C14—C13—H13	119.7	N2—C31—H31	110.5
C15—C14—C13	119.6 (2)	C32—C31—H31	110.5
C15—C14—H14	120.2	C30—C31—H31	110.5
C13—C14—H14	120.2	C31—C32—C18	105.32 (17)
C14—C15—C16	120.8 (3)	C31—C32—H32A	110.7
C14—C15—H15	119.6	C18—C32—H32A	110.7
C16—C15—H15	119.6	C31—C32—H32B	110.7
C15—C16—C11	119.3 (2)	C18—C32—H32B	110.7
C15—C16—H16	120.4	H32A—C32—H32B	108.8
C11—C16—H16	120.4	O7—C33—O8	123.1 (2)
O4—C17—C8	108.33 (18)	O7—C33—C18	124.2 (2)
O4—C17—C18	106.63 (16)	O8—C33—C18	112.65 (19)
C8—C17—C18	116.62 (18)	O8—C34—H34A	109.5
O4—C17—H17	108.3	O8—C34—H34B	109.5
C8—C17—H17	108.3	H34A—C34—H34B	109.5
C18—C17—H17	108.3	O8—C34—H34C	109.5
C33—C18—C32	112.46 (18)	H34A—C34—H34C	109.5
C33—C18—C17	109.74 (17)	H34B—C34—H34C	109.5
C32—C18—C17	115.58 (18)	C10—N1—C1	134.0 (2)
C33—C18—C19	113.03 (18)	C10—N1—C8	96.14 (18)
C32—C18—C19	102.27 (16)	C1—N1—C8	129.89 (19)
C17—C18—C19	103.27 (16)	C19—N2—C31	106.95 (16)
N2—C19—C27	117.35 (18)	C19—N2—C28	119.41 (18)
N2—C19—C20	109.40 (17)	C31—N2—C28	104.83 (18)
C27—C19—C20	102.52 (17)	C4—O1—C7	118.1 (2)
N2—C19—C18	108.18 (17)	C11—O3—C9	115.24 (17)
C27—C19—C18	115.38 (18)	C20—O4—C17	106.50 (16)
C20—C19—C18	102.71 (16)	C20—O5—H5A	109.5
O5—C20—O4	108.25 (18)	C33—O8—C34	116.94 (19)
			
C6—C1—C2—C3	−0.3 (4)	C22—C21—C26—C27	178.8 (2)
N1—C1—C2—C3	−179.3 (2)	C20—C21—C26—C27	−2.2 (3)
C1—C2—C3—C4	0.5 (4)	C24—C25—C26—C21	0.5 (4)
C2—C3—C4—C5	−0.3 (4)	C24—C25—C26—C27	−179.7 (2)
C2—C3—C4—O1	179.5 (2)	C21—C26—C27—O6	167.0 (2)
O1—C4—C5—C6	−179.8 (2)	C25—C26—C27—O6	−12.8 (4)
C3—C4—C5—C6	0.0 (4)	C21—C26—C27—C19	−11.7 (3)
C4—C5—C6—C1	0.2 (4)	C25—C26—C27—C19	168.5 (2)
C2—C1—C6—C5	0.0 (4)	N2—C19—C27—O6	−39.2 (3)
N1—C1—C6—C5	179.1 (2)	C20—C19—C27—O6	−159.0 (2)
N1—C8—C9—O3	−125.71 (19)	C18—C19—C27—O6	90.2 (3)
C17—C8—C9—O3	118.4 (2)	N2—C19—C27—C26	139.48 (19)
N1—C8—C9—C10	−3.95 (16)	C20—C19—C27—C26	19.6 (2)
C17—C8—C9—C10	−119.8 (2)	C18—C19—C27—C26	−91.2 (2)
O3—C9—C10—O2	−63.1 (4)	N2—C28—C29—C30	−34.4 (3)
C8—C9—C10—O2	−179.2 (3)	C28—C29—C30—C31	16.9 (3)
O3—C9—C10—N1	120.4 (2)	C29—C30—C31—N2	7.0 (3)
C8—C9—C10—N1	4.26 (18)	C29—C30—C31—C32	−107.5 (2)
C16—C11—C12—C13	0.4 (4)	N2—C31—C32—C18	35.7 (2)
O3—C11—C12—C13	179.4 (2)	C30—C31—C32—C18	150.7 (2)
C11—C12—C13—C14	0.1 (4)	C33—C18—C32—C31	−144.90 (19)
C12—C13—C14—C15	−0.3 (4)	C17—C18—C32—C31	88.0 (2)
C13—C14—C15—C16	0.1 (4)	C19—C18—C32—C31	−23.4 (2)
C14—C15—C16—C11	0.3 (4)	C32—C18—C33—O7	−139.0 (2)
C12—C11—C16—C15	−0.6 (4)	C17—C18—C33—O7	−8.9 (3)
O3—C11—C16—C15	−179.6 (2)	C19—C18—C33—O7	105.8 (3)
N1—C8—C17—O4	−69.7 (2)	C32—C18—C33—O8	43.1 (3)
C9—C8—C17—O4	29.6 (3)	C17—C18—C33—O8	173.25 (19)
N1—C8—C17—C18	170.06 (18)	C19—C18—C33—O8	−72.1 (2)
C9—C8—C17—C18	−90.6 (2)	O2—C10—N1—C1	0.4 (5)
O4—C17—C18—C33	144.22 (17)	C9—C10—N1—C1	177.1 (2)
C8—C17—C18—C33	−94.7 (2)	O2—C10—N1—C8	178.8 (3)
O4—C17—C18—C32	−87.3 (2)	C9—C10—N1—C8	−4.47 (19)
C8—C17—C18—C32	33.8 (3)	C2—C1—N1—C10	−25.0 (4)
O4—C17—C18—C19	23.5 (2)	C6—C1—N1—C10	156.0 (3)
C8—C17—C18—C19	144.57 (18)	C2—C1—N1—C8	157.1 (2)
C33—C18—C19—N2	124.31 (19)	C6—C1—N1—C8	−21.9 (3)
C32—C18—C19—N2	3.2 (2)	C17—C8—N1—C10	123.5 (2)
C17—C18—C19—N2	−117.18 (18)	C9—C8—N1—C10	4.47 (19)
C33—C18—C19—C27	−9.4 (2)	C17—C8—N1—C1	−58.0 (3)
C32—C18—C19—C27	−130.54 (19)	C9—C8—N1—C1	−177.0 (2)
C17—C18—C19—C27	109.1 (2)	C27—C19—N2—C31	151.62 (19)
C33—C18—C19—C20	−120.06 (19)	C20—C19—N2—C31	−92.2 (2)
C32—C18—C19—C20	118.79 (17)	C18—C19—N2—C31	18.9 (2)
C17—C18—C19—C20	−1.6 (2)	C27—C19—N2—C28	33.0 (3)
N2—C19—C20—O5	−24.3 (3)	C20—C19—N2—C28	149.1 (2)
C27—C19—C20—O5	100.9 (2)	C18—C19—N2—C28	−99.7 (2)
C18—C19—C20—O5	−139.07 (18)	C32—C31—N2—C19	−33.9 (2)
N2—C19—C20—O4	93.87 (19)	C30—C31—N2—C19	−155.86 (18)
C27—C19—C20—O4	−140.89 (17)	C32—C31—N2—C28	93.8 (2)
C18—C19—C20—O4	−20.9 (2)	C30—C31—N2—C28	−28.1 (2)
N2—C19—C20—C21	−145.69 (18)	C29—C28—N2—C19	158.7 (2)
C27—C19—C20—C21	−20.4 (2)	C29—C28—N2—C31	39.0 (3)
C18—C19—C20—C21	99.57 (19)	C5—C4—O1—C7	168.1 (3)
O5—C20—C21—C26	−106.8 (2)	C3—C4—O1—C7	−11.7 (4)
O4—C20—C21—C26	130.4 (2)	C12—C11—O3—C9	140.2 (2)
C19—C20—C21—C26	14.8 (2)	C16—C11—O3—C9	−40.7 (3)
O5—C20—C21—C22	72.0 (3)	C10—C9—O3—C11	96.4 (2)
O4—C20—C21—C22	−50.8 (3)	C8—C9—O3—C11	−162.82 (18)
C19—C20—C21—C22	−166.4 (2)	O5—C20—O4—C17	157.98 (16)
C26—C21—C22—C23	1.0 (4)	C21—C20—O4—C17	−77.3 (2)
C20—C21—C22—C23	−177.8 (2)	C19—C20—O4—C17	37.2 (2)
C21—C22—C23—C24	0.2 (4)	C8—C17—O4—C20	−164.82 (16)
C22—C23—C24—C25	−1.0 (4)	C18—C17—O4—C20	−38.6 (2)
C23—C24—C25—C26	0.6 (4)	O7—C33—O8—C34	−6.0 (4)
C22—C21—C26—C25	−1.3 (4)	C18—C33—O8—C34	171.9 (2)
C20—C21—C26—C25	177.6 (2)		

### Hydrogen-bond geometry (Å, º)

*Cg*7 is the centroid of the C11–C16 ring.

**Table:**

*D*—H···*A*	*D*—H	H···*A*	*D*···*A*	*D*—H···*A*
O5—H5*A*···N2	0.82	2.10	2.638 (2)	122
C15—H15···O6^i^	0.93	2.56	3.231 (3)	129
C16—H16···O2	0.93	2.59	3.405 (3)	146
C32—H32*B*···O3	0.97	2.55	3.413 (3)	148
C12—H12···*Cg*7^ii^	0.93	2.95	3.775 (3)	149

Symmetry codes: (i) *x*−1, *y*, *z*; (ii) −*x*+1, *y*+1/2, −*z*+1/2.

## References

[bb1] Arun, M., Joshi, S. N., Puranik, V. G., Bhawal, B. M. & Deshmukh, A. R. A. S. (2003). *Tetrahedron*, **59**, 2309–2316.

[bb2] Banik, B. K. & Becker, F. F. (2000). *Tetrahedron Lett.* **41**, 6551–6554.

[bb3] Bernstein, J., Davis, R. E., Shimoni, L. & Chang, N.-L. (1995). *Angew. Chem. Int. Ed. Engl.* **34**, 1555–1573.

[bb4] Brakhage, A. A. (1998). *Microbiol. Mol. Biol. Rev.* **62**, 547–585. 10.1128/mmbr.62.3.547-585.1998 PMC98925 9729600

[bb5] Bruker (2008). *APEX2*, *SAINT* and *SADABS* Bruker AXS Inc., Madison, Wisconsin, USA.

[bb6] Farrugia, L. J. (1997). *J. Appl. Cryst.* **30**, 565.

[bb7] Sheldrick, G. M. (2008). *Acta Cryst.* A**64**, 112–122. 10.1107/S0108767307043930 18156677

[bb8] Spek, A. L. (2009). *Acta Cryst.* D**65**, 148–155. 10.1107/S090744490804362X PMC2631630 19171970

